# Gig Work, Telework, Precarity, and the Pandemic

**DOI:** 10.1177/00027642231155371

**Published:** 2023-02-14

**Authors:** Jeremy Schulz, Laura Robinson, Noah McClain, Bianca C. Reisdorf

**Affiliations:** 1 Institute for the Study of Societal Issues, UC Berkeley, Berkeley, CA, USA; 2 Department of Sociology, Harvard Berkman Klein Center, Santa Clara University, Santa Clara, CA, USA; 3 Department of Sociology, Santa Clara University, Santa Clara, CA, USA; 4 Department of Communication Studies, University of North Carolina at Charlotte, Charlotte, NC, USA

**Keywords:** platform economy, gig work, remote work, telework, COVID-19

## Abstract

This issue examines technology-driven economic developments during the global COVID-19 pandemic in Europe, Asia, and the Americas. Specifically, the articles cover the ways that gig work, the platform economy, and remote work have evolved during the course of the pandemic. The issue leads with articles that chart the interplay of the platform economy with various facets of the pandemic from the inequalities and risks faced by gig workers to market forces shaping the commercialization of hosting platforms. The following articles concentrate on the ways in which specific structural conditions—digital infrastructure as well as the structure of the economy—influence the unequal distribution of telework in Uruguay and the relationship between informality and remote work opportunities across Latin America. The last two articles explore remote work in Asia and North America. In the first of these two articles remote work in Japan is examined in order to investigate the cultural sources of resistance to the adoption of remote work. In the second and concluding article, the remote work preferences of U.S. adults are analyzed as a function of technology usage (videoconferencing versus instant messaging) as well as sociodemographic and occupational attributes.

## Technology and Economy During the Covid-19 Pandemic

During the initial stages of the COVID-19 pandemic, the first large-scale pandemic to take place in the digital age, global lockdowns have shifted the role of technology in work and the economy for many. During this time, visible trends in multiple countries suggest that, at least in developed economies, both the platform economy and remote work can be harnessed to harmonize consumption and work with various public health restrictions. However, neither the platform economy nor remote work has proven to be a panacea. This issue of the *American Behavioral Scientist* examines these technology-driven economic developments during the global COVID-19 pandemic in the United States, Europe, Asia, and Latin America. With respect to the platform economy, authors show that the COVID-19 crisis has prompted the platform economy to become more heavily commercialized and capitalistic, despite early promises of democratization pre-pandemic. At the same time, different types of platform economy workers continue to find themselves different levels of health risk that combined with preexisting economic precarity in complex ways. For some platform economy workers at the mercy of an often exploitative and volatile platform economy, both economic precarity and health risks can vary significantly, depending on both economic factors and the health situation. As far as remote work is concerned, while the pandemic has undoubtedly turbocharged the adoption of remote work across the developed world—particularly among white collar workers—it has also come up against various structural, sociodemographic, organizational, and cultural limits. While the structural limits of remote work are most visible in developing economies, both sociodemographic and organizational constraints are also evident in countries of the Global North, including the United States and Japan. Thus, the contributions in this issue all suggest that the ongoing digitization of the economy is an uneven process, even as it has been accelerated by the pandemic in particular contexts and settings.

## The Platform Economy

We begin the issue with an extended discussion of the interrelationships between the platform economy, health risks due to the COVID-19 pandemic, and economic risks for workers due to labor oversupply, precarious work arrangements, and instability in consumer demand. Written by Paola Tubaro and Antonio Casilli, this lead article delves into these interconnections as they concern three distinctive types of digitally mediated work: object-and person-jobs mediated through online platforms (e.g., delivery jobs), fully online labor, and what they call “social networking labor” performed on behalf of social media platforms. Whereas the first type of work takes place in physical space and thus does little to minimize health risks, the latter two types do reduce such risks. After examining these three forms of digitally mediated work, the article concludes that online workers who work on digitized content continued to bear economic risks associated with labor oversupply, even as they faced far fewer risks to their health as contrasted with workers who performed some kind of physical labor. Thus, as Tubaro and Casilli demonstrate, platformization of work—whether physical or nonphysical labor—does not in itself contain either health risks or economic risks in the pandemic and post-pandemic world.

In the second piece on the platform economy, we turn to the case of the platform-based market for residential rentals mediated through Airbnb. Airbnb has long been considered one of the platforms anchoring the digitally enabled platform economy, which ostensibly serves to connect producer–consumers through depersonalized peer-to-peer networks. In this contribution, the authors Mehmet Cansoy and Juliet Schor probe the role of commercialized actors on the Airbnb site during the first stage of the COVID-19 pandemic in U.S. rental markets. They find that, contrary to expectations, the crisis amplified the precrisis trend whereby professionalized renters displaced the casual renters, which formed the basis of the “peer-to-peer” model which Airbnb pioneered. In fact, the pandemic crisis saw the growing dominance of commercialized renters as opposed to small-scale “mom-and-pop” renters. This trend is observed even in U.S. cities which implemented strict regulations designed specifically to constrain the commercialization of the platform. Thus, as Cansoy and Schor show, even during a crisis, which was expected to weaken the power of commercialized actors on Airbnb, the mediation of rental markets through this centralized platform has actually strengthened the relative position of commercialized actors.

## Remote Work and Work From Home

Next, we turn from the platform economy to remote work and telework in Latin America. We start with an article examining the state of remote work in Uruguay. The study by Matías Dodel and María Julia Acosta analyzes survey data to account for the relative prevalence of remote work among different workers across the Uruguayan private and public sectors. The models they introduce characterize remote work adoption as a function of both the workers’ individual-level attributes in combination with the organizational-level attributes of employers and the employers’ sectoral locations. Statistical estimations demonstrate that, although individual-level characteristics account for a large proportion of the variation in remote work uptake, the strongest predictor of such uptake is the sectoral location of the employer and specifically whether the employing organization has formalized pandemic-related remote work policies. This is followed by another predictor of remote work uptake, namely the extent to which workers have access to digital infrastructures and resources. These findings point to the key roles of both the public sector and organizational acceptance of remote work within the case study of Uruguay.

We next look at the structural features of Latin American economies that may either facilitate or hinder remote work on the part of various sociodemographic and occupational groups. This article, authored by Daniela de los Santos and Inés Fynn, draws on macro-level survey data regarding the potential for remote work across different groups in seven Latin American countries. Their analysis reveals that the potential for remote work varies strongly across countries and within countries, according to sociodemographic and occupational axes of differentiation. Across all countries, female-dominated occupations offer a greater potential for remote work than male-dominated occupations. Yet even as informal work plays an important role across these Latin American countries, most informal work does not lend itself to remote work solutions, as it involves the manipulation of physical goods and/or face-to-face interactions. Workers in the informal economy also tend to live in conditions that are not conducive to working from home. Moreover, workers in the informal economy typically lack the social protections afforded to those in the formal economy. What this means, in the context of the restrictions introduced in the wake of the pandemic, is that informal workers are a large and particularly disadvantaged subgroup within the workforce. As the authors reveal, informal workers can neither take advantage of remote work options to safeguard health nor depend on social protections to combat the economic insecurity brought about by unemployment and precarious work. Thus, in the situation of the pandemic, informal workers face a double burden of increased health risks and heightened economic insecurity.

Whereas remote work faces a number of structural and socioeconomic barriers to adoption in Latin American countries, it is culture that best explains the puzzle posed by Japan’s non-adoption of remote work during the pandemic. When compared to peer countries, remote work did not become nearly as widespread in Japan after the onset of the pandemic, despite the highly developed IT infrastructure of the country and the large portion of workers who worked in teleworkable jobs. Hiroshi Ono addresses this puzzle by exploring the distinctive culture of the Japanese workplace and its emphasis on face-to-face interactions and physical copresence. As Ono reveals, the Japanese office workplace favors physical copresence and the manipulation of paper documents over remote work and digital artifacts to a far greater degree than other countries. Significantly, even when remote work is efficient and productive, these cultural conventions have a strong grip across the Japanese economy for a number of reasons. Further, as Ono argues, the ritualistic norm of physical copresence stems from the importance of visible status hierarchies within the Japanese workplace, as well as the preference for pervasive “micromanagement” of lower ranking employees by supervisors. As the article shows, the worldwide trend toward more remote work thus confronts unusual obstacles in Japan.

As the final contribution in the issue makes clear, by contrast, remote workers in the United States face a different landscape. Leveraging nationally representative survey data from U.S. adults in the early part of the pandemic, Jeremy Schulz, Øyvind Wiborg, and Laura Robinson analyze the intention to work from home. This study inquires into the behavioral, sociodemographic, occupational, and technological sources of differences in the desire to work from home during the hypothetical future. The piece identifies the impacts of two common workplace communication platforms alongside other potential determinants of work from home intentions, including pre-pandemic and pandemic work from home behaviors, sociodemographic backgrounds, and socioeconomic/occupational attributes. The analysis finds that the frequency of text messaging (e.g., Slack) and the frequency of videoconferencing (e.g., Zoom) have diametrically opposed effects on intentions to work from home in the future: whereas a higher frequency of text messaging is associated with a stronger preference for frequent work-from-home, the frequency of videoconferencing exerts the opposite effect on preferences. We propose several potential mechanisms to explain this inverse association, including the differential usage of these two types of platforms across different occupations, differences in attentional and affective demands of the two platforms, and the relative work autonomy enjoyed by the types of workers who are most likely to rely on these platforms.

In closing, by bringing together research from Europe, Asia, Latin America, and North America, this issue provides rich insights into technology-driven economic developments during the global COVID-19 pandemic. Matching the global reach of contributions, the research assembled here probes the interplay of the platform economy with various facets of the pandemic from the inequalities and risks faced by gig workers to market forces shaping the commercialization of hosting platforms. In terms of telework, several contributions bring to light the ways in which specific structural conditions—digital infrastructure as well as the structure of the economy—influence the unequal distribution of telework in developing economies. Contributions focusing on developed economies highlight challenges to remote work from cultural sources of resistance to telework to differentiation in telework preferences as a function of digital technology usage. Across these articles, readers acquire new insights into the ways that gig work, the platform economy, and remote work have evolved during the course of the pandemic.

